# Latent Tuberculosis Patients Have an Increased Frequency of IFN-γ-Producing CD5+ B Cells, Which Respond Efficiently to Mycobacterial Proteins

**DOI:** 10.3390/pathogens12060818

**Published:** 2023-06-09

**Authors:** Julio Flores-Gonzalez, Lucero A. Ramón-Luing, Jesus Romero-Tendilla, Alexia Urbán-Solano, Alfredo Cruz-Lagunas, Leslie Chavez-Galan

**Affiliations:** 1 Laboratory of Integrative Immunology, Instituto Nacional de Enfermedades Respiratorias Ismael Cosio Villegas, Mexico City 14080, Mexico; jfloresg1707@alumno.ipn.mx (J.F.-G.); ramonluing@yahoo.com.mx (L.A.R.-L.); jeusromerotendilla@gmail.com (J.R.-T.); alexursol25@outlook.com (A.U.-S.); 2 Laboratory of Immunobiology and Genetic, Instituto Nacional de Enfermedades Respiratorias Ismael Cosio Villegas, Mexico City 14080, Mexico; alfredocl@gmail.com

**Keywords:** B cells, latent tuberculosis (LTB), IFN-γ-producing, CD5+ B cells

## Abstract

Tuberculosis (TB) remains a public health problem worldwide and is one of the deadliest infectious diseases, only after the current COVID-19 pandemic. Despite significant advances in the TB field, there needs to be more immune response comprehension; for instance, the role played by humoral immunity is still controversial. This study aimed to identify the frequency and function of B1 and immature/transitional B cells in patients with active and latent TB (ATB and LTB, respectively). Here we show that LTB patients have an increased frequency of CD5+ B cells and decreased CD10+ B cells. Furthermore, LTB patients stimulated with mycobacteria’s antigens increase the frequency of IFN-γ-producing B cells, whereas cells from ATB do not respond. Moreover, under the mycobacterial protein stimulus, LTB promotes a pro-inflammatory environment characterized by a high level of IFN-γ but also can produce IL-10. Regarding the ATB group, they cannot produce IFN-γ, and mycobacterial lipids and proteins stimulate only the IL-10 production. Finally, our data showed that in ATB, but not in LTB, B cell subsets correlate with clinical and laboratory parameters, suggesting that these CD5+ and CD10+ B cell subpopulations have the potential to be biomarkers to differentiate between LTB and ATB. In conclusion, LTB has increased CD5+ B cells, and these cells can maintain a rich microenvironment of IFN-γ, IL-10, and IL-4. In contrast, ATB only maintains an anti-inflammatory environment when stimulated with mycobacterial proteins or lipids.

## 1. Introduction

Respiratory diseases affect the airways and lungs, impacting lung capacity and gas distribution in our body; they represent more than 7% of mortality rates worldwide [1,2]. Tuberculosis (TB) is an infectious disease that mainly affects the lung and is caused by the bacillus *Mycobacterium tuberculosis* (Mtb).

Globally, TB remains a leading cause of death and an infectious disease worldwide. The last report from the World Health Organization (WHO) estimated that 10.6 million people developed ATB; 89% of cases occurred in men (57%) and women (32%) older than 15 years and 11% in infants [3].

The clinical status of active TB (ATB) is one of many problems to solve; however, controlling latent TB (LTB) is necessary for TB eradication. LTB is an infection caused by Mtb, where the immune response contains the pathogen. However, it is not eliminated, and the patient has an absence of any clinical manifestation of the disease. Approximately 5–10% of LTB individuals will develop ATB at some point, mainly associated with immunosuppression [4,5].

It is known that professional antigen-presenting cells, such as monocytes, macrophages, and B cells, are the main niche of infection and the first cellular line of defense and activation of adaptive immune responses for the control and elimination of Mtb [6]. However, the role of humoral immunity, mediated by B cells, against Mtb infection is still controversial. Nevertheless, evidence indicates that B cells play a significant role during the TB immune response [7,8]. For instance, B cells in the lung granuloma present antigens to T cells, secrete antibodies, and modulate inflammation [9].

Reports indicate that persons infected with Mtb have circulating antibodies against Mtb antigens, which could play an essential role in pathogenesis or protection [10]. In addition, following Mtb antigens stimulation, there are B cells that are a source of polyfunctional cytokine responses such as interleukin (IL)-10, IL-4, and granulocyte-macrophage colony-stimulating factor (GM-CSF) [11]. Moreover, these cells also produce IFN-γ to favor macrophage polarization [12].

Diverse B cell subpopulations have been described. For instance, CD5+ B cells, also known as B1 cells, are an innate-like B cell population responsible for producing natural antibodies, rapid humoral immunity, and immune regulation [13]. A remarkable CD5+CD1d+ B cell subset that produces IL-10 and suppresses T-cell responses also has been reported [14,15]. CD10+ B cells are another subpopulation described in humans, considered immature/transitional B, an early emigrant from the bone marrow [16]. This subset is expanded in advancing viral infections and associated with increased levels of IL-7 [17]; unfortunately, little is known about the role of this B cell population in LTB vs. ATB.

A comprehensive knowledge of the immune response during ATB vs. LTB is necessary to accomplish the goal of TB control. Here, we analyzed the frequency, phenotype, and function of circulating CD5+ and CD10+ B subsets from ATB and LTB. Our data suggest that CD5+ B cells accumulated in LTB subjects play a previously unrecognized role; these cells promote interferon-gamma (IFN-γ) production under Mtb-protein stimulus, contrary to the hyporesponsive state observed in ATB.

## 2. Materials and Methods

### 2.1. Ethics Statement

This study was approved by the Institutional Ethics Committee of the Instituto Nacional de Enfermedades Respiratorias (B06–22) in Mexico City, Mexico. All participants signed written informed consent forms. All procedures were performed in agreement with the 1964 Helsinki Declaration and the ethical standards of the Institutional Ethics Committees.

### 2.2. Study Populations

**Figure 1:**
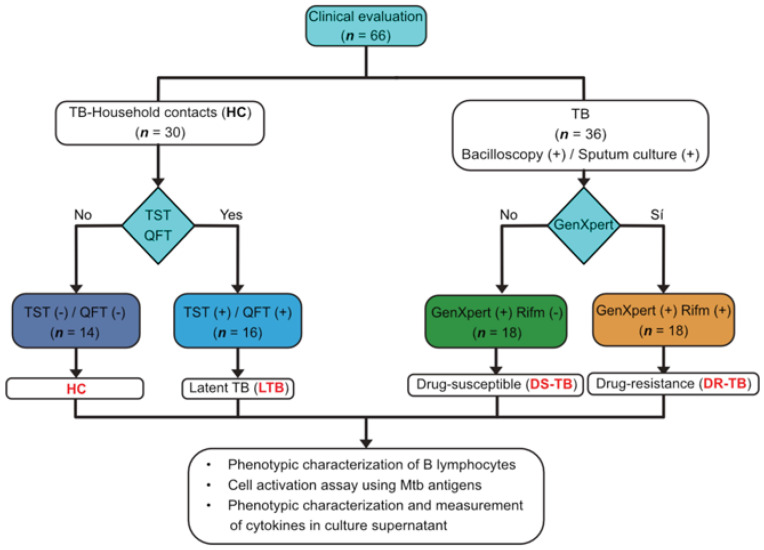
Workflow of enrolled patients. A clinical evaluation of subjects > 18 years old was performed in this study. Thirty household contacts (HC) were enrolled and stratified into either uninfected contact (UC, *n* = 14) who were negative by both tuberculin skin test (TST) and QuantiFERON-Gold In-Tube (QFT) or latently infected contacts (LTB, *n* = 16) who were positive by both TST and QFT. Thirty-six subjects were diagnosed with ATB based on bacilloscopic and sputum culture positive results. Then, they were stratified into drug-susceptible Mtb (DS-TB, *n* = 18) and drug-resistant (DR-TB, *n* = 18) based on GenXpert results. In addition, mononuclear cells from some patients were used for cell phenotype by flow cytometry (UC, *n* = 8; LTB, *n* = 10; DS-TB, *n* = 10, and DR-TB, *n* = 10), and others were used to evaluate the cell activation by Mtb antigens and then, phenotypic characterization and measurement of cytokines in culture supernatant [UC, *n* = 4; LTB, *n* = 4; DS-TB, *n* = 5, and DR-TB, *n* = 5]. QFT: QuantiFERON-Gold In-Tube test; TST: tuberculin skin test; PCR Xpert MTB/RIF: polymerase chain reaction Xpert *Mycobacterium tuberculosis*/rifampicin.

A total of 36 subjects > 18 years old with a TB diagnosis were recruited for this study. They were stratified into drug-susceptible Mtb (DS-TB, *n* = 18) and drug-resistant (DR-TB, *n* = 18) based on GenXpert results and Mtb culture isolation and drug sensitivity testing (Figure 1, right). Notably, 94% (17 out of 18) of DR-TB patients received a TB diagnostic (at least two years before the current) and a treatment previously, but it was discontinued, suggesting that DR-TB patients display a chronic status of an anti-TB immune response, whereas in DS-TB patients, the current TB diagnostic was for the first time; they were naïve to treatment. Symptoms’ severity and duration were not different between DR-TB and DS-TB groups.

A total of 30 household contacts (HC) were evaluated and stratified into either uninfected contact (UC, *n* = 14) who were confirmed negative by both QuantiFERON-Gold In-Tube (QFT) and tuberculin skin test (TST) or latently infected contacts (LTB, *n* = 16) who were confirmed positive by both TST and QFT (Figure 1, left). TST and QFT tests were performed in HC when they and their ATB patients were enrolled (at diagnosis time). HC is defined as an individual who shared the same enclosed living space for at least one night/week or extended periods during the day with the ATB patient for three months before the TB diagnostic [18].

All subjects were enrolled during 2016–2018 at the Instituto Nacional de Enfermedades Respiratorias, Mexico City, Mexico. Patients with HIV infection, cancer, chronic obstructive pulmonary disease, solid-organ transplant recipients, and those undergoing immunosuppressive or anticoagulant therapy were excluded.

### 2.3. Cells

Circulating blood samples were obtained at diagnosis time, before the initiation of anti-TB therapy. Plasma and peripheral blood mononuclear cells (PBMCs) were recovered using BD Vacutainer tubes (BD Biosciences, Franklin Lakes, NJ, USA). PBMCs were isolated by standard Lymphoprep^TM^ (Accurate Chemical-Scientific, Westbury, NY, USA) centrifugation gradient within one hour of the blood draw and were subsequently cryopreserved. Plasma was obtained and stored at −70 °C until use.

### 2.4. Multiparametric Flow Cytometry Analysis

PBMCs were prepared to evaluate cell surface marker expressions using monoclonal antibodies (mAbs) to CD3, CD19, CD5, CD10, and CD1d. All the mAbs were provided by BioLegend (San Diego, CA, USA). The cells used for Fluorescence Minus One (FMO) condition were stained and acquired in parallel to identify background levels of staining; dead cells were omitted using viability staining Zombie Red Dye solution (BioLegend). More details of the antibodies used can be found in Table S1.

The data were acquired using a FACS Aria II flow cytometer (BD Biosciences, San Jose, CA, USA) equipped with FACSDiva 6.1.3 software (BD Biosciences). In each condition, at least 50,000 events were acquired per sample. The flow cytometry data file (FCS) was analyzed using Flow Jo™ v10.6.1 (Flow Jo, LLC, Ashland, OR, USA). Before the analysis strategy, a quality control analysis using flowAI was made to detect and remove anomalies from FCS data analyzing the flow rate, signal acquisition, and dynamic range in each sample acquired [19]. After flowAI analysis, the analysis strategy consisted of limiting the singlet cells through forward scatter (FSC-A vs. FSC-H), and viability plots were selected (Zombie Red negative). Finally, the frequency (percentage) of B cells (CD19+ cells) was identified and classified as CD20− or CD20+ cells (Figure S1).

### 2.5. PBMC’s Stimulation

Amounts of 5 × 10^5^ PBMCs/mL of UC (*n* = 4), LTB (*n* = 4), DS-TB (*n* = 5), and DR-TB (*n* = 5) were plated in RPMI-1640 medium supplemented with 2 mM L-glutamine, 1M HEPES (Gibco™, Grand Island, NY, USA), penicillin–streptomycin–amphotericin B solution (Gibco™), and 10% fetal bovine serum (Gibco™). Each sample was cultured with 10 mg/mL of total protein (TP) or total lipids (TL) obtained from Mtb, provided by BEI Resources, NIAID, NIH: Mycobacterium tuberculosis, strain H37Rv (TP: NR-14831 and TL: NR-14837) at 37 °C, 5% CO_2_. After 72 h of culture, the cellular fraction was collected for flow cytometry staining, and supernatants were stored for analysis of cytokines production. Unstimulated PBMCs were cultured as a negative control.

### 2.6. Quantification of Intracellular IFN-γ and IL-10, upon In Vitro Stimulation with Mtb H37Rv Antigens

Four hours before the end of incubation, 1 μg/mL of brefeldin A (bfA) (BD Biosciences) was added. The cellular fraction was stained with a cocktail of mAbs anti-CD3, CD19, CD5, and CD23 (Biolegend). After a wash with cell staining buffer (Biolegend), cells were permeabilized with BD Cytofix/Cytoperm™ buffer (BD Biosciences) for 20 min at 4 °C. Cells were washed and incubated in the dark for 30 min at 4 °C with mAbs anti-IL-10 and IFN-γ (Biolegend). Finally, the cells were washed, resuspended in cell staining buffer (BioLegend), and kept at 4 °C until acquisition.

The data were acquired using a FACS Aria II flow cytometer (BD Biosciences) equipped with FACSDiva 6.1.3 software (BD Biosciences). In each condition, at least 50,000 events were acquired per sample. The flow cytometry data file (FCS) was analyzed using Flow Jo™ v10.6.1 (Flow Jo, LLC). More details of the antibodies used can be found in Table S1.

### 2.7. LEGENDplex^TM^ Assay

The LEGENDplex kits used were multiplex bead-based assay panels manufactured by BioLegend. The bead panels that were chosen for measurement of cytokine concentration in every sample included the pro-inflammatory cytokines IFN-γ and the anti-inflammatory cytokines IL-4 and IL-10. The bead assays were performed following instructions provided by the manufacturer. More details of the kit used can be found in Table S1.

### 2.8. Spearman Correlation Matrix

The Spearman correlation matrix was produced in R v4.2.0 using the corrplot version 0.92 package. The color and size of the circles in the diagram represent the magnitude and direction of the correlation. The *p*-values were also calculated, and the data points with *p*-values larger than 0.05 are not shown in the diagram, indicating a lack of statistical confidence.

### 2.9. Statistical Analysis

Data are shown as median values and interquartile ranges (IQR, 25–75). The D’Agostino–Pearson test was used to test the normality of data. Kruskal–Wallis’s test with Dunnett’s post-test was used for multiple comparisons; *p* < 0.05 were considered statistically significant. Statistical analysis was performed using GraphPad Prism V 9.0.2 (GraphPad Software, Inc., San Diego, CA, USA).

## 3. Results

### 3.1. Clinical Characteristics of the Study Population

The demographic and clinical characteristics of patients are summarized in Table 1. UC and DS-TB had a lower median age than LTB (UC, *p* < 0.0027; DS-TB, *p* < 0.0099), and gender was no different between groups. ATB groups had a lower BMI than UC (DS-TB, *p* < 0.0041; DR-TB, *p* < 0.0138) and LTB (DS-TB, *p* < 0.0001; DR-TB, *p* < 0.0005). Regarding the Bacillus Calmette–Guérin (BCG) vaccine, 78–93% of patients received it during childhood.

The drug sensitivity test showed that 100% of DS-TB patients were sensitive to first-line drug to TB, whereas 100% of DR-TB patients were rifampicin-resistant but also showed resistance to another first-line drug. Additionally, five DR-TB patients showed resistance to moxifloxacin and ofloxacin, which are second-line drugs to TB (Table S2).

Diabetes mellitus type 2 was a more frequent comorbidity in the DR-TB group compared to UC (*p* < 0.001), LTB (*p* < 0.005), or DS-TB (*p* < 0.014). Although a high frequency of hypertension has been reported in TB patients [20], this condition was not different between groups.

Peripheral leukocytosis was observed in both ATB groups; DS-TB had a higher absolute count of circulating total leukocytes than UC (*p* < 0.0059) and LTB (*p* < 0.0348), whereas DR-TB had a higher count than UC (*p* < 0.0327). In addition, the monocyte count was also higher in both DS-TB and DR-TB than UC (*p* < 0.0055, *p* < 0.0092, respectively) and LTB (*p* < 0.0004, *p* < 0.0077, respectively). Similarly, the neutrophil count was higher in DS-TB than in UC (*p* < 0.0001) and LTB (*p* < 0.0019), whereas DR-TB displayed a higher count than UC (*p* < 0.0251).

In consonance with a previous report [21], the DS-TB group showed a higher platelet count than UC (*p* < 0.0001) and LTB (*p* < 0.0001). Similarly, DS-TB patients had a higher count of platelets than DR-TB patients (*p* < 0.0004).

Finally, biochemical blood parameters showed that DR-TB patients had higher glucose levels than UC (*p* < 0.0193).

In summary, ATB groups presented lower BMI than UC and LTB. Notably, DR-TB patients showed high percentages of type 2 diabetes with high glucose levels, whereas LTB showed a profile of circulating immune cells like UC, and ATB groups showed a profile close to inflammation.

### 3.2. The Frequency of CD5+ B Cells Is Higher in LTB Than in ATB

**Figure 2:**
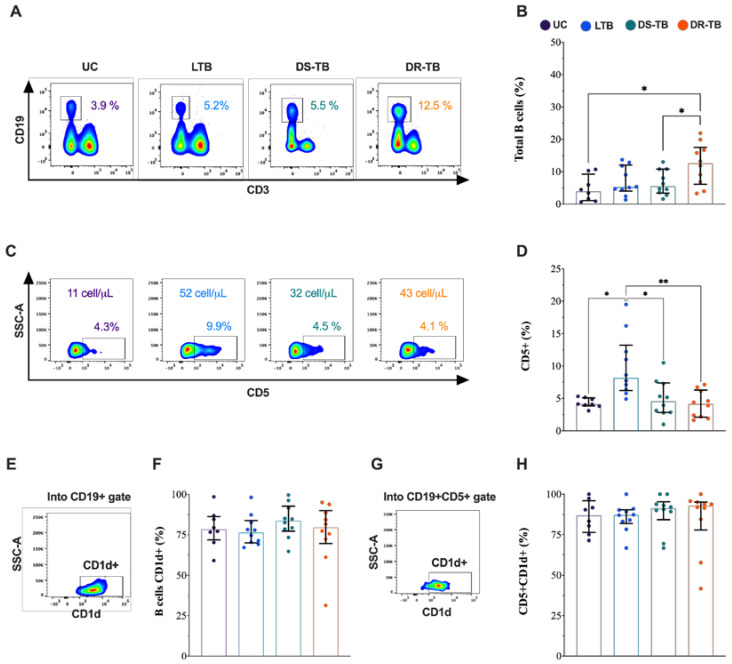
High frequency of CD5+ B cells in LTB subjects. (**A**) Representative dot plot of the CD19+ cells gate evaluated by flow cytometry in each TB group. The median is shown inside the plot. (**B**) Frequency of B cells between groups. (**C**) Representative density plots per group of the CD5 expression inside the B cells gate. The median and total number of CD5+ B cells/mL of blood are shown inside the plot. (**D**) Frequency of CD5+ B cells, comparison between all TB groups. (**E**) Representative density dot plot of CD19+CD1d+ cells from an LTB patient. (**F**) Frequency of CD19+CD1d+ cells between groups. (**G**) Representative density dot plot of CD5+CD1d+ B cells from an LTB patient. (**H**) Frequency of CD5+CD1d+ B cells, comparison between all TB groups. Data are shown as median with interquartile range (IQR, 25–75). The statistical comparison was performed using Kruskal–Wallis’s test, * *p* < 0.05, ** *p* < 0.01. UC: uninfected contact (*n* = 8); LTB: latent tuberculosis (*n* = 10); DS-TB: drug-susceptible Mtb (*n* = 10); DR-TB: drug-resistant (*n* = 10).

Our first aim was to evaluate the frequency of B cell (CD19+) subpopulations by flow cytometry (Figure 2A). DR-TB showed an increased frequency of B cells [13 (6–18)] compared to HD and DS-TB [4 (1–9), *p* < 0.0155; 5 (3–11), *p* < 0.0411; respectively] (Figure 2B). Into the CD19+ gate, the expression of CD5 was evaluated (Figure 2C), and LTB showed a higher frequency of CD19+CD5+ cells [10 (6–13)], compared to UC [4 (4–5), *p* < 0.0170], DS-TB [5 (3–7), *p* < 0.0408], and DR-TB [4 (2–6), *p* < 0.0040] (Figure 2D). The CD5 expression (FMI) on B cell showed a low CD5 expression on UC [950 (872–1003)] compared to LTB [1372 (1266–1831), *p* < 0.0002], DS-TB [1219 (1130–1400), *p* < 0.0293], and DR-TB [1231 (1167–1324), *p* < 0.0230] (Figure S2A).

Due the increased frequency of CD5+ B cells in LTB, we took the blood cells count reported by the clinical laboratory, and we obtained the total number of CD5+ B cells/mL of blood. Data showed that LTB [52 (22–84), *p* < 0.0203] and DR-TB [43 (11–66), *p* < 0.0205] showed a higher number of CD5+ count compared to UC [11 (2–16)] (Figure S2B).

The frequency of the CD19+CD1d+ and CD19+CD1d+CD5+ subsets were evaluated (Figure 2E,G, respectively). We could not identify differences between TB groups in the frequency of B cells positive to CD1d (Figure 2F) and CD19+CD1d+CD5+ cells (Figure 2H). Data suggest that general B cell frequencies are increased in DR-TB patients, but the CD5+ B cells subpopulation is exclusively increased during LTB.

### 3.3. The CD10+ B Cells Frequency Is Decreased in LTB and DR-TB

**Figure 3:**
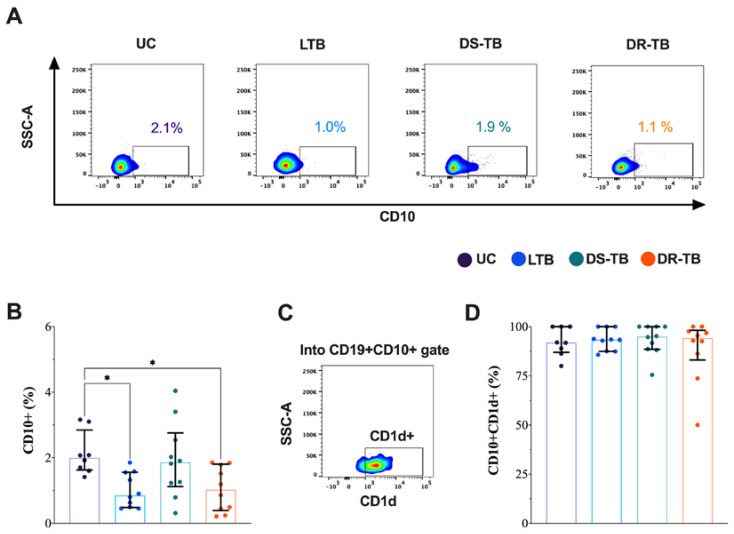
The frequency of CD10+ B cells is decreased during LTB. (**A**) Representative density dot plots of B cells expressing CD10 with the median in each study group. The median is shown inside the plot. (**B**) Frequency of CD10+ B cells, comparison between all TB groups. (**C**) Representative density dot plot of CD10+CD1d+ B cells from an LTB patient. (**D**) Frequency of CD10+CD1d+ B cells, comparison between all TB groups. Data are shown as median with interquartile range (IQR, 25–75). The statistical comparison was performed using Kruskal–Wallis’s test, * *p* < 0.05. UC: uninfected contact (*n* = 8); LTB: latent tuberculosis (*n* = 10); DS-TB: drug-susceptible Mtb (*n* = 10); DR-TB: drug-resistant (*n* = 10).

Following this, the frequency of the CD19+CD10+ B cells subset, considered transitional B cells, was evaluated (Figure 3A).

The frequency of CD10+ B cells was lower in LTB [0.9 (0.5–2)] compared to UC [2 (2–3), *p* < 0.0278]. Similarly, DR-TB patients showed lower CD10+ B cells frequencies [1 (0.4–2)] compared to UC (*p* < 0.0278), but DS-TB patients did not change (Figure 3B). The CD1d expression on the CD10+ B cells was evaluated (Figure 3C), but it is not different between TB groups (Figure 3D).

Together, these findings indicate that LTB and DR-TB decrease the frequency of transitional B cells in the peripheral blood.

### 3.4. DR-TB Exhibits a Complex Network of Correlations between B Immunological Profile and Clinical Outcome

LTB had a different profile of CD5+ and CD10+ B cell subsets compared to ATB, suggesting that both B cell subsets could be associated with the infection state by Mtb. Therefore, we performed correlograms to determine if B cell subsets impact clinical parameters. 

**Figure 4:**
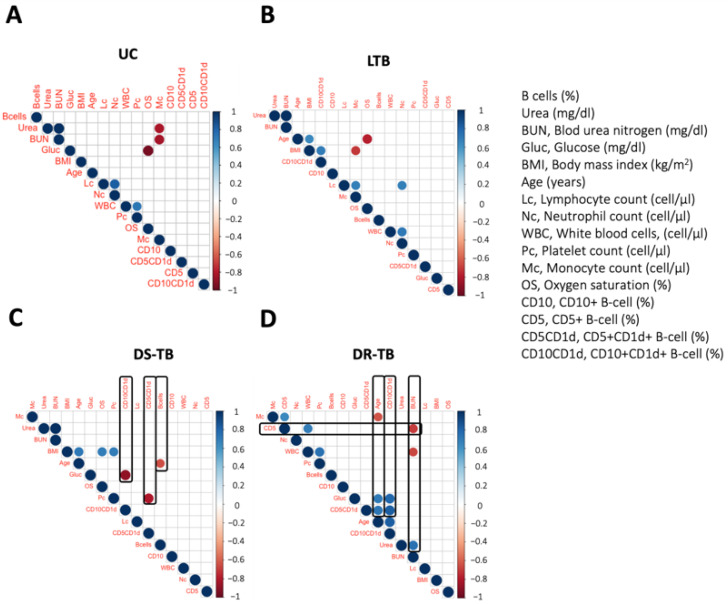
Spearman correlation matrix of the proportion of the B cells populations among the studied TB groups. Correlation between clinical parameters and B cells frequencies subpopulations in UC volunteers (**A**), LTB subjects (**B**), DS-TB (**C**), and DR-TB (**D**) patients. The blue color indicates a strong positive correlation, and the red indicates a strong negative correlation. Black boxes show associations with B cells phenotypes. The size and color intensity of the dots are proportional to the Spearman correlation coefficients (*rs*). UC: uninfected contact (*n* = 8); LTB: latent tuberculosis (*n* = 10); DS-TB: drug-susceptible Mtb (*n* = 10); DR-TB: drug-resistant (*n* = 10).

The TB spectrum shows a different degree of complexity in the correlation network; UC has a minimal number of correlations; among them, the positive correlations were between lymphocyte count and neutrophil count (*rs* = 0.82; *p* < 0.0116), and white blood cells and platelet count (*rs* = 0.72; *p* < 0.043); however, these groups also showed negative correlations between glucose and oxygen saturation (*rs* = −0.95; *p* < 0.0002), and monocytes and BUN (*rs* = −0.83; *p* < 0.010) (Figure 4A). 

The LTB group has positive correlations between white blood cells and neutrophil count (r = 0.72; *p* < 0.0189), lymphocyte count and neutrophil count (*rs* = 0.69; *p* < 0.027), lymphocyte count and monocyte count (*rs* = 0.68; *p* < 0.0299), body mass index (BMI) correlated with age (*rs* = 0.64; *p* < 0.046), and CD10+CD1d+ B cells (*rs* = 0.69; *p* < 0.026) (Figure 4B). Conversely, negative correlations were observed between monocyte count and BMI (*rs* = −0.70; *p* < 0.023) and between oxygen saturation and age (*rs* = −0.77; *p* < 0.008) (Figure 4B).

DS-TB showed positive correlations between BMI with three parameters: age (*rs* = 0.71; *p* < 0.0205), oxygen saturation (*rs* = 0.70; *p* < 0.024), and platelet count (*rs* = 0.70; *p* < 0.024) (Figure 4C). Moreover, negative correlations were observed between CD10+CD1d+ and glucose (*rs* = −0.88; *p* < 0.0007), CD5+CD1d+ B cells with platelet count (*rs* = −0.80; *p* < 0.005), and B cell frequency with age (*rs* = −0.65; *p* < 0.042) (Figure 4C).

DR-TB showed a great diversity of correlations; among positives, we observed the CD5+ B cells frequency correlated with WBC (*rs* = 0.70; *p* < 0.031) and with monocytes (*rs* = 0.64; *p* < 0.045), and white blood cells and platelet count (*rs* = 0.72; p < 0.043). Similarly, the CD10+CD1d+ B cells frequency correlated with three parameters: glucose (*rs* = 0.80; *p* < 0.005), age (*rs* = 0.83; *p* < 0.003), and CD5+CD1d+ B cells frequency (*rs* = 0.82; *p* < 0.003). In addition, age has a positive correlation with blood glucose (*rs* = 0.71; *p* < 0.022) and CD5+CD1d+ B cells frequency (*rs* = 0.72; *p* < 0.0180) (Figure 4D). Finally, our data indicate an age-associated negative correlation with monocyte count (*rs* = −0.64; *p* < 0.047), and two negative correlations between BUN and CD5 frequency cells (*rs* = −0.70; *p* < 0.03), and white blood cells (*rs* = −0.66; *p* < 0.044) (Figure 4D). 

These correlations show that UC and TBL groups have low correlations, and apparently, the B cell subsets do not influence the clinical parameters. However, ATB showed clinical-parameters-associated correlations with B cells phenotypes, noting that DR-TB has a complex network that appears to control clinical parameters. 

### 3.5. LTB Increases the Frequency of IFN-γ-Producing CD5+ B Cells in Response to Proteins from Mtb

**Figure 5:**
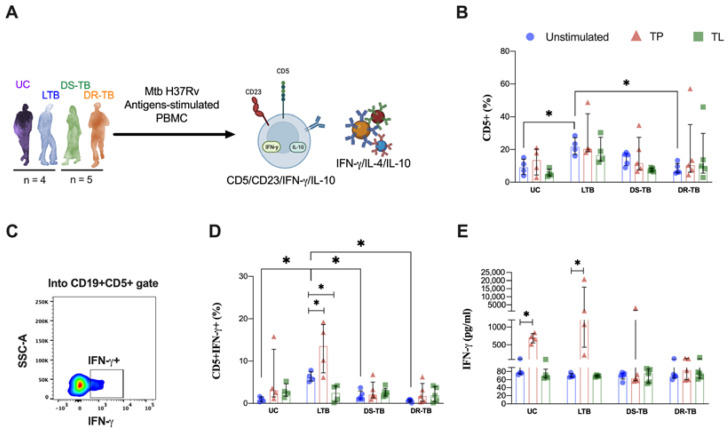
TP induces IFN-γ secretion by CD5+ B cell during LTB. (**A**) PBMCs from four groups, comprising UC, LTB, DS-TB, and DR-TB, were stimulated for three days with total proteins (TP, orange triangle) or total lipids (TL, green square) from Mtb H37Rv. The unstimulated condition was included as a control stimulation (blue circle). Cells were analyzed by flow cytometry. (**B**) Analysis of CD5+ B cell frequency. (**C**) Representative density dot plot of CD5+IFN-γ+ B cells from an LTB patient. (**D**) The frequency of cytokine-positive cells out of CD5+ B cells for each stimulation was analyzed for IFN-γ. (**E**) IFN-γ supernatant levels. Data are represented as median and IQR values. Statistical comparisons were performed by Kruskal–Wallis’s test, * *p* < 0.05. UC: uninfected contact (*n* = 4); LTB: latent tuberculosis (*n* = 4); DS-TB: drug-susceptible Mtb (*n* = 5); DR-TB: drug-resistant (*n* = 5).

The frequency of the CD5+ B cell subpopulation is modified between LTB and ATB. To clarify this subset’s functional capacity, we evaluated its ability to respond to proteins (TP) or lipids (TL) from Mtb H37Rv. By flow cytometry, intracellular IFN-γ and IL-10 were assessed in the CD5+ B cell subset, whereas, in the culture, supernatant IFN-γ, IL-4, and IL-10 were measured (Figure 5A).

Similar to what is observed in Figure 2D, the frequency of CD5+ B cells is higher in LTB at baseline [22 (17–27)] compared to both UC [9 (5.0–14.1), *p* < 0.0426] and DR-TB [6 (6–12), *p* < 0.0144]; however, this frequency is not modified even with TP or TL stimuli (Figure 5B). Next, by flow cytometry, we clarified if, although the frequency is not altered, they can produce IFN-γ (Figure 5C).

Data showed that LTB group has a higher frequency of IFN-γ-producing CD5+ B cells at baseline [6 (5–8)] compared to UC [0.9 (0.4–1.5), *p* < 0.0355], DS-TB [1 (0.9–3), *p* < 0.0159] and DR-TB [0.8 (0.3–0.8), *p* < 0.0022]. Moreover, LTB cells stimulated with TP increased the frequency of IFN-γ-producing CD5+ B cells compared to its unstimulated condition [14 (7–19), *p* < 0.045]; conversely, the TL stimuli decreased IFN-γ-producing CD5+ B cells compared to its unstimulated condition [2 (0.4–4), *p* < 0.0286]. The ATB group did not modify the frequency of this subset under any stimuli condition (Figure 5D).

In consonance with the frequency of IFN-γ-producing CD5+ B cells in the presence of TP stimulus, LTB produces a high level of IFN-γ when PBMCs were stimulated with TP [unstimulated: 68 (67–75); TP: 1208 (432–1601), *p* < 0.0307], and a similar result was observed in the UC group [unstimulated: 77 (74–102); TP: 699 (557–821), *p* < 0.0286] (Figure 5E).

Thus, LTB has a high frequency of CD5+ B cells, which are IFN-γ-producing. Moreover, the culture microenvironment is rich in IFN-γ, suggesting that the CD5+ B cell subset is significant during LTB to activate IFN-γ-dependent mechanisms of control.

### 3.6. LTB Produces High Levels of IL-10 and IL-4 in Response to Proteins and Lipids from Mtb, but It Is Independent of the B Cells Subpopulations

**Figure 6:**
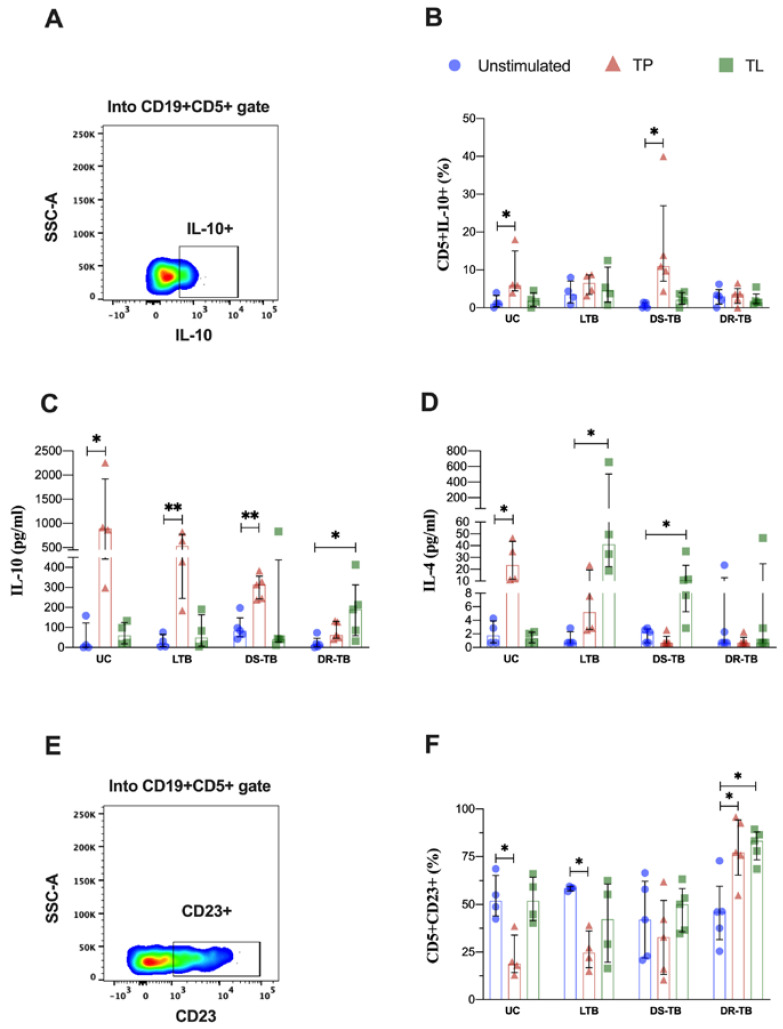
TP induces IL-10 secretion by CD5+ B cells in UC subjects and DS-TB patients. (**A**) Representative density dot plot of CD5+IFN-γ+ B cells from an LTB patient. (**B**) IL-10-producing CD5+ B cells frequency. (**C**,**D**) IL-10 and IL-4 supernatant levels, respectively. (**E**) Representative density dot plot of CD5+CD23+ B cells from an LTB patient. (**F**) The frequency of CD5+CD23+ B cells. TP stimulus is shown as the orange triangle, TL as a green square, and the unstimulated condition is shown as a blue circle. Data are represented as median and IQR values. Statistical comparisons were performed by Kruskal–Wallis’s test, * *p* < 0.05, ** *p* < 0.01. UC: uninfected contact (*n* = 4); LTB: latent tuberculosis (*n* = 4); DS-TB: drug-susceptible Mtb (*n* = 5); DR-TB: drug-resistant (*n* = 5).

LTB increases the IFN-γ-producing CD5+ B cell subset frequency in response to TP stimulus. Then, we evaluated the capacity of this subset to produce the anti-inflammatory cytokine IL-10 (Figure 6A). UC increased the frequency of IL-10-producing CD5+ B cells with TP stimulus compared to the unstimulated condition [unstimulated: 1 (0.3–3.4); TP: 6 (5–15), *p* < 0.0477]; DS-TB induced a similar profile [unstimulated: 0.4 (0–1); TP: 11 (7–27), *p* < 0.0222]. LTB and DR-TB groups did not show changes (Figure 6B).

Consequently, anti-inflammatory cytokines were evaluated in the culture supernatant, and TP increased the IL-10 levels produced by PBMCs in UC [unstimulated: 6 (0.6–122); TP: 893 (442–1922), *p* < 0.0247], LTB [unstimulated 18 (4–65); TP: 536 (246–775), *p* < 0.0046], and DS-TB groups [unstimulated: 84 (54–148); TP: 314 (242–356), *p* < 0.0079]. To note, the DR-TB group increased IL-10 levels with the LT stimulus but not with TP [unstimulated: 12 (2–45); TL: 190 (59–313), *p* < 0.0242] (Figure 6C). 

Regarding IL-4 levels, UC increased the level with TP stimulus [unstimulated: 2 (0.7–4); TP: 24 (12–44), *p* < 0.0151]. Interestingly, LTB increased IL-4 levels but under TL stimulus [0.7 (0.7–2); TL: 41 (22–504), *p* < 0.0106]; the DS-TB group was similar [unstimulated: 2 (0.7–3); TL: 11 (5–23), *p* < 0.0461]. Regarding DR-TB, there were no observed changes (Figure 6D).

CD23 has been described as a marker of activated B cells; however, its expression on CD5+ B cells remains unexplored in a TB infection context. Thus, we evaluated the CD23 expression on B cells, and it did not show differences (Figure S2A), following the CD23 expression assessed on the CD5+ B subset (Figure 6E). Despite the IL-10 and IL-4 production by the CD5+ B cell subset, the frequency of the CD5+CD23+ subpopulation was decreased in UC and LTB patients stimulated with TP [UC: unstimulated, 52 (44–65); TP, 19 (14–34), *p* < 0.0285; LTB: unstimulated, 59 (57–59); TP, 25 (17–36), *p* < 0.0372]. On the contrary, DR-TB patients increased the CD5+ CD23+ B cells frequency in both TP and TL stimuli [unstimulated: 46 (32–60); TP: 77 (65–94), *p* < 0.0324; TL: 83 (73–88), *p* < 0.0267] (Figure 6F). The expression of CD23 into the B cells producer of IFN-γ and IL10 was not modified (Figure S3B,C, respectively).

These results suggest that IL-10 and IL-4 are produced differentially between the TB spectrum; while LTB produces IL-10 in response to TP and IL-4 in response to TL, ATB groups respond differently between them.

## 4. Discussion

B cells are essential to both innate and adaptative immunity. In contrast to the T cells that require an antigen-presenting cell (APC) to be activated, B cells can be activated by direct interaction with the pathogen and are the mediator of the humoral response [22].

In the TB context, the role of antibodies is controversial, and various studies have focused on monocyte–macrophage reprogramming as the primary APC [6,23]. However, evidence shows that Mtb alters both phenotype and function, inclusive; it is suggested that Mtb reprograms hematopoietic stem cells to limit myelopoiesis via IFN-I response to induce cell death [24].

Recently, it was reported that LTB and ATB have no differences in the frequency of circulating B cells. However, that study did not clarify if the ATB group is drug-sensitive or -resistant [25]. This characteristic is relevant because our data showed that LTB and DS-TB have a similar frequency of B cells; however, DR-TB has increased frequency, suggesting that the active disease could play a role in maintaining the high frequency of B cells.

Indeed, initial contact with the mycobacteria promotes circulating B cells that display altered phenotype and function, and it is present in LTB and ATB. However, after successful anti-TB treatment, it is normalized [26]. We observed that LTB and ATB had altered the frequency of B cell subsets, but only in ATB do these subsets correlate with clinical parameters, and it is stronger in DR-TB than DS-TB.

Our study has the limitation that we did not evaluate the B cells distribution after anti-TB treatment. However, previously we demonstrated that DR-TB still exhibits a systemic inflammatory status even with six months of treatment, whereas DS-TB normalizes this status [27]. Here it is also necessary to mention that previously we demonstrated that even with 16 months of anti-TB therapy, DR-TB fails to normalize the distribution of some immune cells. In contrast, DS-TB is able to normalize the distribution with six months of treatment [28]. Thus, evaluating the B cell subsets distribution after a successful anti-TB therapy is necessary, but the differences between DS-TB and DR-TB should be considered.

Other studies reported an elevated frequency of B cells. However, these results were obtained from TB patients infected with Mtb strains resistant to isoniazid, rifampicin, streptomycin, and pyrazinamide [29,30], and our DR-TB group included patients resistant to rifampicin plus another first-line drug to TB. We suggested that the number of resistances acquired by the mycobacteria also plays an essential role in modulating the immune response.

This study demonstrated that the CD5+ B cell subset is increased in LTB compared to HD and ATB. Moreover, LTB increases the frequency of IFN-γ-producing CD5+ B cells in response to proteins from Mtb, suggesting that the subpopulation CD5+ cells play an important role during the defense against Mtb, probably by the production of IFN-γ, a cytokine that is well-known for its capacity to activate the bactericide mechanism [31,32].

Other reports also demonstrated that during mycobacterial infections, there is an expansion of the CD5+ B cell subpopulation [33,34,35]. This is according with the CD5+ count reported in this study to LTB and DR-TB patients. However, contrary to frequency, the CD5 expression does not decline progressively as the disease progresses from LTB to DS-TB and DS-TB to DR-TB. This suggests that the decline of CD5 frequency may not be associated with loss of anti-TB immunity because B cells maintained a constant CD5 expression. However, an increase in the population could confirm this phenomenon.

Furthermore, these cells have been reported as clones that display highly selective binding specificities, and they produce polyreactive antibodies [36]; in this regard, it has been observed that B cells exposed to Mtb lipids increase the non-specific anti-phospholipid IgM secretion [37].

Evidence supports that LTB has a different profile of antibodies titer compared to ATB and even compared to recently infected individuals [38,39]. Under a cancer context, it is known that STAT3 signaling regulates the CD5 expression [40], but in the TB context, this signaling has not been clarified; further studies are necessary to identify the molecules involved to favor the expansion of CD5+ B cells in LTB.

Contrary to our data, a previous study has shown an increase in CD1d+ CD5+ B cells in ATB patients to suppress the immune response [14]. Furthermore, reports indicate that CD1d expression on APC could be modified by the cytokines’ microenvironment [41], there are probably differences between both study groups—for instance, time with symptoms, Mtb strain, and inflammatory status, among others—and this has an impact on regulating the presence of the CD1d+CD5+ B cells.

Here, we reported for the first time that CD10+ B cells, also called peripheral blood transitional, are decreased in LTB and DR-TB patients, suggesting that Mtb favors B cell modulation. Probably, the gradual loss of CD10+ B cells observed in LTB and DR-TB patients is a consequence of the generation of B cell subsets from stimulated transitional cells, as previously proposed [42]. Similarly, reports suggest that B cells develop in a linear pathway, which means that this development could be from immature cells in the bone marrow or from transitional cells in the periphery [43]. This will confirm the increased B cells observed and which are related to the pressure exerted by Mtb infection and the progress of the disease.

However, since it is a population with less frequency in circulation, it is necessary to implement animal models where the biological functions of these cells can be better explored, especially in the context of IL-7 remodeling, as previously demonstrated [17].

To confirm the capacity of B cell subsets to respond to Mtb antigens, we stimulated PBMCs with TP and TL from Mtb, and data showed that during ATB, the CD5+ B cells have a hyporesponsive state, which is more prominent in DR-TB. On the one hand, LTB responded to the TP stimulus by producing IFN-γ and IL-10, whereas the TL stimulated the IL-4 production. On the other hand, DR-TB only can produce IL-10 in response to lipids, and DS-TB produces IL-10 in response to TP and IL-4 to TL. Together, this suggests that LTB efficiently responds to lipids or proteins from Mtb and can maintain the production of pro- and anti-inflammatory cytokines. In contrast, ATB only can maintain an anti-inflammatory status.

Diverse studies have demonstrated the relevance of IFN-γ during TB; it induces the transcription of more than 200 genes in macrophages to eliminate Mtb; however, some bacterial antigens, such as the 19 kDa lipoprotein, block the transcription of genes [44]. As previously reviewed, these CD5+ B cells could be considered IFN-γ-producing innate B cells [45].

Some reports indicate that IL-4 is elevated in cavitary granuloma formation in ATB patients [46], but others suggest that IL-4 is low in circulating B cells from TB patients [47]. We observed that in vitro, the UC PBMCs produce high levels of IL-4 in response to TP, but LTB and DS-TB only produce IL-4 in response to lipids. Considering that IL-4 is a crucial cytokine to induce the immunoglobulin class-switching [48], our results open a new question regarding the relevance of the CD5+ B cells to favor the pro- and anti-inflammatory profile and evaluate if the IL-4 induced by proteins (like in UC) or by lipid (like in LTB and DS-TB) use the same mechanisms of regulation.

Thus, our present study provides new insight into the regulation of CD5+ B cell responses, particularly of IFN-γ-responses, in patients with LTB. However, further investigations are warranted to dissect the mechanism underlying the activity of CD19+ CD5+ B cells as well as the exact molecule (s) mediating such a function.

## 5. Conclusions

The results show that CD5+ B cells are dispensable for the control of TB. LTB has a high frequency of circulating CD5+ B cells, and under Mtb proteins stimulus, they produce IFN-γ, whereas ATB cannot produce this relevant cytokine. However, the altered frequency of B cell subsets correlates with clinical and laboratory parameters, suggesting that these CD5+ and CD10+ B cell subpopulations can be biomarkers to differentiate between LTB and ATB.

## Figures and Tables

**Table 1:** Demographic and clinical characteristics of patients.

Variable	UC [A] (*n* = 14)	LTB [B] (*n* = 16)	DS-TB [C] (*n* = 18)	DR-TB [D] (*n* = 18)	*p*-Value
Demographic characteristics ^1^					
Age, years	28 (22–41)	50 (45–57)	36 (25–42)	45 (34–59)	* [A vs. B] * [B vs. C]
Male, *n* (%) ^2^	10 (67)	6 (37)	8 (44)	12 (67)	ns
Female, *n* (%) ^2^	5 (33)	10 (63)	10 (56)	6 (33)	ns
Body mass index, kg/m^2^	27 (23–28)	30 (25–33)	21 (19–24)	22 (18–25)	** [A vs. C] * [A vs. D] *** [B vs. C] *** [B vs. D]
Oxygen saturation, %	95 (91–95)	95 (92–98)	96 (94–96)	95 (90–97)	ns
BCG vaccine, *n*^2^	13	14	15	15	ns
Clinical characteristics, *n* (%) ^2^					
Coexisting conditions					
Type 2 diabetes	0 (0)	1 (7)	3 (17)	10 (56)	*** [A vs. D] ** [B vs. D] ** [C vs. D]
Hypertension Dis.	0 (0)	2 (13)	0 (0)	2 (11)	ns
Blood cell count (×10^3^ cell/μL) ^1^					
White blood cells	6 (5–7)	7 (6–8)	9 (7–12)	8 (6–10)	** [A vs. C] * [A vs. D] * [B vs. C]
Lymphocyte count	2 (2–3)	2 (2–3)	2 (1–2)	2 (1–2)	ns
Monocyte count	0.4 (0.1–0.5)	0.4 (0.4–0.5)	0.6 (0.5–0.9)	0.6 (0.5–0.9)	** [A vs. C] ** [A vs. D] *** [B vs. C] ** [B vs. D]
Neutrophil count	3 (2–4)	4 (3–5)	6 (5–8)	5 (3.0–9)	*** [A vs. C] * [A vs. D] ** [B vs. C]
Platelet count	238 (225–307)	241 (222–275)	478 (365–514)	280 (195–384)	**** [A vs. C] *** [B vs. C] ** [C vs. D]
Biochemical blood parameters, mg/dL ^1^					
Glucose	94 (86–102)	98 (94–104)	98 (92–149)	133 (90–150)	* [A vs. D]
Urea	22 (17–27)	21 (18–28)	20 (16–23)	19 (17–25)	ns
Blood urea nitrogen	10 (8–13)	10 (8–13)	9 (7–11)	9 (7–11)	ns

^1^ Kruskal–Wallis’s test with Dunn’s multiple comparison test. ^2^ Chi-squared tests. * *p* < 0.05, ** *p* < 0.01, *** *p* < 0.001, **** *p* < 0.0001. ns = not significant.

## Data Availability

The original contributions presented in the study are included in the article/Supplementary Material. Further inquiries can be directed to the corresponding author.

## Supplementary Materials

The following supporting information can be downloaded at: https://www.mdpi.com/article/10.3390/pathogens12060818/s1, Table S1: Antibodies used for flow cytometry and ELISA; Table S2: Mycobacterium tuberculosis phenotypic drug susceptibility testing of patients with ATB; Figure S1: General flow cytometry strategy; Figure S2: Expressions of CD5 and total number of CD5+ B cells/mL of blood are increased in TB patients; Figure S3: CD23 frequency on CD5+ B cells.
