# Supplementary material for: Latent Tuberculosis Patients Have an Increased Frequency of IFN-γ-Producing CD5+ B Cells, Which Respond Efficiently to Mycobacterial Proteins
Source: Pathogens. 2023 Jun 9;12(6):818. doi: 10.3390/pathogens12060818 (PMC10304593; doi:10.3390/pathogens12060818)
Supplement: Supplementary file 1 [file pathogens-12-00818-s001.zip › pathogens-2404608-supplementary.pdf]

pathogens

MDPI

Article

Latent tuberculosis patients have an increased frequency of IFN-γ-producing CD5+ B cells, which respond efficiently to mycobacterial proteins

Julio Flores-Gonzalez [1], Lucero A. Ramón-Luing [1], Jesus Romero-Tendilla [1], Alexia Urbán-Solano [1], Alfredo Cruz-Lagunas [2], Leslie Chavez-Galan [1,*]

[1] Laboratory of Integrative Immunology, Instituto Nacional de Enfermedades Respiratorias Ismael Cosio Villegas, Mexico City 14080, Mexico; jfloresg1707@alumno.ipn.mx (J.F.-G.); ramonluing@yahoo.com.mx (L.A.R.-L.); jeusromerotendilla@gmail.com (J.R.-T.); alexursol25@outlook.com (A.U.-S.)

[2] Laboratory of Immunobiology and Genetic, Instituto Nacional de Enfermedades Respiratorias Ismael Cosio Villegas, Mexico City 14080, Mexico; alfredocl@gmail.com

* Correspondence: lchavez_galan@iner.gob.mx or lchavezgalan@gmail.com; Tel.: +52-5554871700 (ext. 5270)

Supplementary Material

Table S1. Antibodies used for flow cytometry and ELISA.

Antibody	Conjugate to	Clone/Catalog[1]	Company
CD3	APC-Cy7	OKT3	Biolegend
CD3	PB	OKT3	Biolegend
CD19	BV421	HIB19	Biolegend
CD19	APC-Cy7	HIB19	Biolegend
CD5	BV510	L17F12	Biolegend
CD23	PE-Cy7	EBVCS-5	Biolegend
CD10	FITC	HIT10a	Biolegend
CD1d	PE	NA	Biolegend
Viability	PE TexRed	NA	Biolegend
IL-10	AF488	JES3-9D7	Biolegend
IFN-γ	APC	4S.B3	Biolegend
Legendplex™	NA	740267	Biolegend

[1] The clone is indicated for flow cytometry antibodies and the catalog number for Legenplex™. NA, not applicable.

Table S2. Mycobacterium tuberculosis phenotypic drug susceptibility testing of patients with ATB.

Resistance to	Doses µg/ml	DS-TB (n, %)	DR-TB (S or R) (n, %)
		**1st line DST**	
Streptomycin	1		S (9, 50%), R (9, 50%)
	4		S (14, 78%), R (4, 22%)
Isoniazid	1		S (10, 56%), R (8, 44%)
	4		S (11, 61%), R (7, 39%)
Rifampicin	1	S (18, 100%)	R (18, 100%)
Ethambutol	5		S (17, 95%), R (1, 5%)
	8		S (17, 95%), R (1, 5%)
Pyrazinamide	100		S (15, 83%), R (3, 17%)
		**2nd line DST**	
Amikacin	1		S (18, 100%)
	2		S (18, 100%)
kanamycin	2.5		S (18, 100%)
Capreomycin	2.5	NA	S (18, 100%)
	5		S (18, 100%)
Moxifloxacin	1		S (13, 72%), R (5, 28%)
	2		S (13, 72%), R (5, 28%)
Ofloxacin	2		S (13, 72%), R (5, 28%)

DST, drug susceptibility testing; S, sensitive; R, resistant; NA, non-applicable

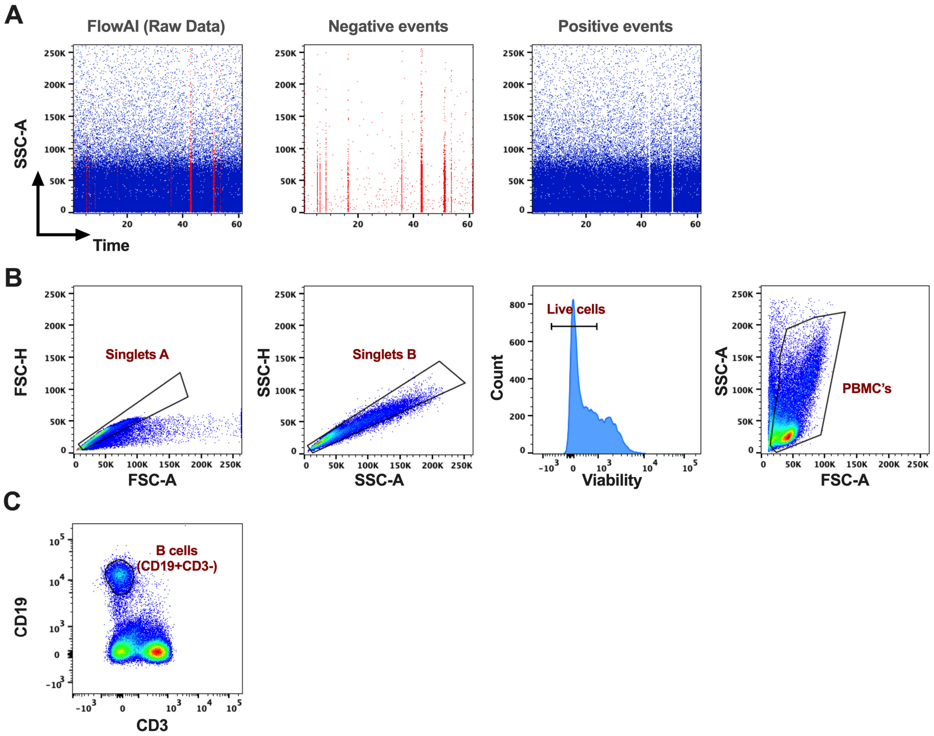

Figure S1. General flow cytometry strategy. Representative results were obtained in B cells sub-populations from TB patients: (**A**) FlowAI analysis was done as a quality control and removed anomalies related to FCS data (negative events). This data cleaning lets evaluated events with common flow rate, signal acquisition, and dynamic range of each sample acquired (positive events). (**B**) Then, viable cells were selected through singlets analysis by forward scatter (FSC-A versus FSC-H) and side scatter (SSC-A versus SSC-H). After that, cell viability was evaluated, and PBMCs were selected. (**C**) Finally, CD19-positive events were named B cells.

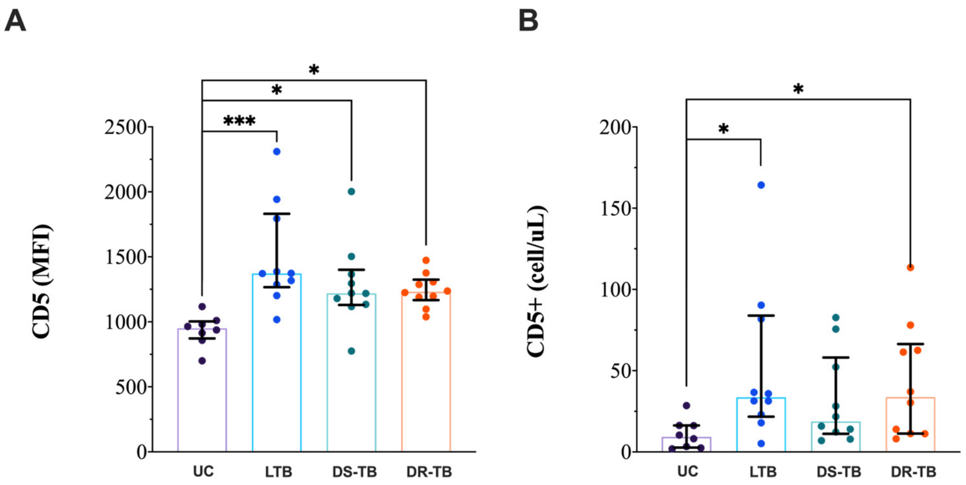

Figure S2. Expressions of CD5 and total number of CD5+ B cells/ml of blood are increased in TB patients. (**A**) CD5 expression (FMI) on B cells between groups. (**B**) Total number of CD5+ B cells/ml of blood between groups. Data are shown as median with interquartile range (IQR, 25–75). The statistical comparison was performed using Kruskal–Wallis's test, * $p < 0.05$, *** $p < 0.001$. UC, uninfected contact (n = 8); LTB, latent tuberculosis (n = 10); DS-TB, drug-susceptible Mtb (n = 10); DR-TB, rifampicin-resistant (n = 10).

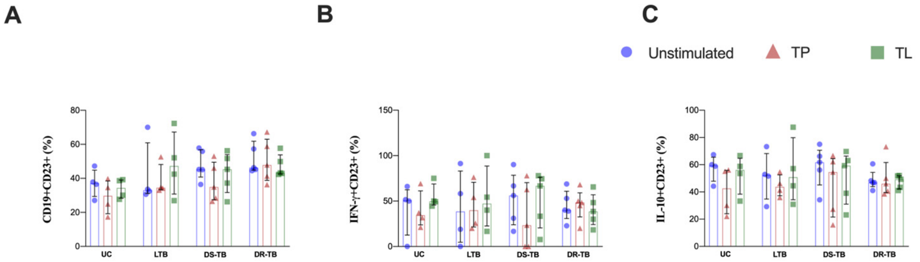

Figure S3. CD23 frequency on CD5+ B cells. (**A**) Frequency of CD19+CD23+ B cells. (**B**) Frequency of CD23+ evaluated on IFN-g-producing CD5+ B cells. (**C**) Frequency of CD23+ IL-10-producing CD5+ B cells. TP stimuli are shown as an orange triangle, TL as a green square, and the unstimulated condition is shown as a blue circle. Data are represented as median and IQR values. Kruskal-Wallis's test performed statistical comparisons. UC, uninfected contact (n = 4); LTB, latent tuberculosis (n = 4); DS-TB, drug-susceptible Mtb (n = 5); DR-TB, rifampicin-resistant (n = 5).
